# Metabolomic screening of pre-diagnostic serum samples identifies association between α- and γ-tocopherols and glioblastoma risk

**DOI:** 10.18632/oncotarget.9242

**Published:** 2016-05-09

**Authors:** Benny Björkblom, Carl Wibom, Pär Jonsson, Lina Mörén, Ulrika Andersson, Tom Børge Johannesen, Hilde Langseth, Henrik Antti, Beatrice Melin

**Affiliations:** ^1^ Department of Chemistry, Umeå University, SE-90187 Umeå, Sweden; ^2^ Department of Radiation Sciences, Oncology, Umeå University, SE-90187 Umeå, Sweden; ^3^ Cancer Registry of Norway, Institute of Population-Based Cancer Research, N-0304 Oslo, Norway

**Keywords:** population-based, serum metabolite, vitamin E, antioxidants, brain tumor

## Abstract

Glioblastoma is associated with poor prognosis with a median survival of one year. High doses of ionizing radiation is the only established exogenous risk factor. To explore new potential biological risk factors for glioblastoma, we investigated alterations in metabolite concentrations in pre-diagnosed serum samples from glioblastoma patients diagnosed up to 22 years after sample collection, and undiseased controls. The study points out a latent biomarker for future glioblastoma consisting of nine metabolites (γ-tocopherol, α-tocopherol, erythritol, erythronic acid, myo-inositol, cystine, 2-keto-L-gluconic acid, hypoxanthine and xanthine) involved in antioxidant metabolism. We detected significantly higher serum concentrations of α-tocopherol (p=0.0018) and γ-tocopherol (p=0.0009) in future glioblastoma cases. Compared to their matched controls, the cases showed a significant average fold increase of α- and γ-tocopherol levels: 1.2 for α-T (p=0.018) and 1.6 for γ-T (p=0.003). These tocopherol levels were associated with a glioblastoma odds ratio of 1.7 (α-T, 95% CI:1.0-3.0) and 2.1 (γ-T, 95% CI:1.2-3.8). Our exploratory metabolomics study detected elevated serum levels of a panel of molecules with antioxidant properties as well as oxidative stress generated compounds. Additional studies are necessary to confirm the association between the observed serum metabolite pattern and future glioblastoma development.

## INTRODUCTION

The etiology of malignant brain tumors is unclear. Commonly known carcinogenic exposures, such as smoking and alcohol consumption, have not been identified as risk factors for glioma [1]. Rare exposures of moderate to high doses of ionizing radiation have been associated with brain tumors and meningioma [2]. On the contrary, asthma and allergies are consistently associated with a reduced risk of glioma, even if the mechanism for this association is poorly understood [3–5]. A familial aggregation of glioma is evident and genomic variations have been characterized and linked to glioma development. Germline genetic mutations, somatic mutations, deletions, and amplifications are known risk factors for glioma development [6–9]. In most cases, the functional mechanisms of how genomic variations initiate tumor development are not known. Nevertheless, brain tumors containing mutated isocitrate dehydrogenase give rise to specific metabolic signatures [10]. Metabolomics, the global study of small molecular compounds and endogenously produced low molecular weight metabolites, can be used to detect and quantify changes in the metabolome. The metabolome reflects all cellular processes and is a direct outcome of gene expression, enzymatic and protein activity. Changes in the metabolome may reflect genomic variations or cellular changes as a result of exogenous exposures, making metabolomics an expanding field in disease biomarker discovery.

We performed an agnostic search, without a prior hypothesis in order to generate novel hypothesis regarding molecular events resulting in glioblastoma development. In this population-based, nested case-control study, we analyzed changes in the metabolic profile of future glioblastoma cases and matched controls. We performed an unbiased global metabolomics screen of pre-diagnostic serum samples from a large set of glioblastoma cases and controls collected up to 22 years before glioblastoma diagnosis. Our metabolomics screen identifies a latent biomarker, indicating an imbalanced redox homeostasis in future glioblastoma cases. Especially elevated tocopherol levels were evident in cases compared to matched controls. This information may be used to generate novel hypothesis regarding molecular events that occur upstream of the metabolome and results in glioblastoma development.

## RESULTS

**Figure 1:**
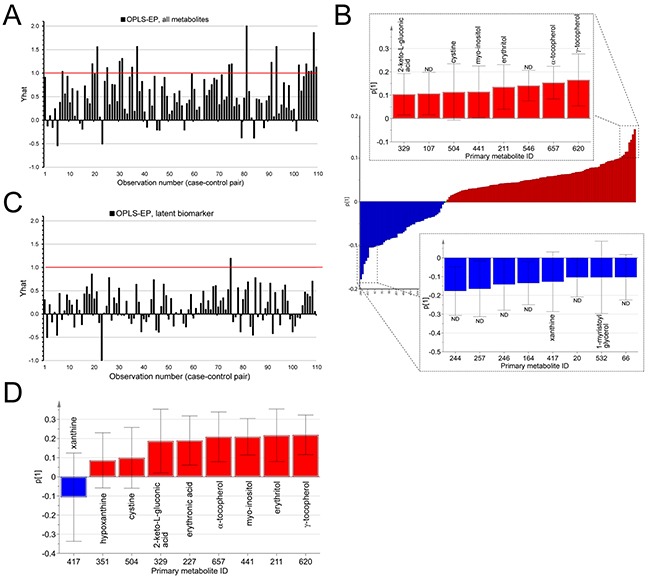
Multivariate statistical analysis of processed GCxGC-TOFMS data by means of OPLS-EP **A.** Bar graph of the estimated effect (Yhat) on the metabolite profile of detected metabolites, reflecting the difference between future glioblastoma case and control over all matched sample pairs (bars in plot). Yhat = 1, corresponds to the target value for the OPLS-EP model. The size of each bar correspond to the dissimilarity of each case-control pair. Small values indicate small differences between the case and control. Large values indicate large differences, and negative values correspond to a difference in the opposite direction between matched case and control. **B.** Bar graph of the predictive loading (p1) for the metabolite pattern associated with future glioblastoma diagnosis. Metabolites with positive loading values in p1 (red) are higher in concentration in future cases, while metabolites with negative loading values in p1 (blue) are lower in concentration. Inset bar graphs highlights the models most important variables, with p1 > 0.1 (red) and p1 < −0.1 (blue). ND = non-determined metabolite. Error bars indicate a 95% confidence interval. **C.** Similar bar graph as in “A”, showing Yhat for the OPLS-EP model containing the metabolite profile of only nine metabolites, latent biomarker, linked to altered antioxidant metabolism. **D.** Bar graph and 95% confidence intervals of the predictive loading (p1) for the latent biomarker, associated with future glioblastoma diagnosis.

To discover compounds associated with future development of glioblastoma, we profiled metabolites in serum samples collected 0.5-22 years before tumor diagnosis. The average time between blood collection and glioblastoma diagnosis was 12.6 years and the average age of the cohort participants was 44.2 years (Table 1). In total, 220 serum samples were metabolically profiled using an unbiased comprehensive GCxGC-TOFMS screening approach. From this, 432 small molecular compounds were detected; 180 confidently identified and annotated with known molecular structures by spectral database comparison (Supplementary Table S1). We applied multivariate analysis in order to extract patterns of metabolites or latent biomarkers, associated with future glioma diagnosis. The data generated OPLS-EP model had a goodness of fit R^2^Y value of 0.54, and a predictive Q^2^ value of 0.21 (Figure 1A). The cross-validated model was highly significant for the difference between matched case and control sample (p = 2.1*10^−7^). The model loadings (weights) revealed that the cases, compared to the controls, had increased levels of γ-tocopherol, α-tocopherol, erythritol, myo-inositol, cystine and 2-keto-L-gluconic acid (Figure 1B). The model also revealed that the cases, compared to the controls, had decreased serum levels of xanthine, 1-myristoyl glycerol and several unidentified metabolites (Figure 1B). Univariate statistical analysis of the identified metabolites for the paired case-control samples showed a statistical significant increase in γ-tocopherol (p = 0.0009), α-tocopherol (p = 0.0018), 2-keto-L-gluconic acid (p = 0.007), erythritol (p = 0.022), N-acetyl-L-alanine (p = 0.031), xylose (p = 0.039) and erythronic acid (p = 0.039) in cases compared to control (Table 2). Interestingly, many of the contributing metabolites were associated with altered antioxidant metabolism, which led us to calculate a separate OPLS-EP model (Figure 1C) including metabolites linked to these mechanisms (i.e. γ-tocopherol, α-tocopherol, erythritol, erythronic acid, myo-inositol, cystine, 2-keto-L-gluconic acid, hypoxanthine and xanthine). The extracted metabolites (i.e. the latent biomarker) in the cross-validated model were highly significant in association with glioblastoma development (p = 5.2*10^−4^) and notably more significant than the individual metabolites included in the model, a finding that suggest a covariation effect (Figure 1D).

**Table 1:** Characteristics of glioblastoma tumor cases and matched controls

Variable	Cases (n=110)	Controls (n=110)
Average age at blood collection, years (SD)	44.2 (7.3)	44.2 (7.2)
Average age at cancer diagnosis, years (SD)	55.9 (8.6)	n/a
Average time from blood collection to diagnosis, years (SD)	12.6 (5.1)	n/a
Date of blood collection, median, calendar years (range)	1990 (1986-1991)	1990 (1986-1991)
Date of birth, median, calendar years (range)	1948 (1923-1955)	1948 (1923-1955)
Male sex, number (%)	82 (74.5%)	82 (74.5%)
Average sample storage time in freezer, years (SD)	24.7 (1.5)	24.7 (1.5)
Blood collection to glioblastoma diagnosis, number/time period		
0–5 years	8	n/a
5–10 years	26	n/a
10–15 years	35	n/a
15–20 years	37	n/a
>20 years	4	n/a

SD, standard deviation, n/a, not applicable

**Table 2:** Summary of significantly altered metabolites

Primary ID	Identification	Match^a^	CAS number	ΔRI^b^	p-value (t-test)	p-value (paired t-test)	mean peak area change (%)^c^
620	γ-tocopherol	911	7616-22-0	3	**0.0008**	**0.0009**	**46**
657	α-tocopherol	848	7695-91-2	1.8	**0.0041**	**0.0018**	**39**
329	2-keto-L-gluconic acid	871	29123-55-5	13.3	**0.0109**	**0.0070**	10
211	erythritol	936	149-32-6	1.8	0.0506	**0.0221**	8
125	N-acetyl-L-alanine	723	1115-69-1	1.1	0.1414	**0.0314**	5
285	xylose	915	58-86-6	8	0.0566	**0.0386**	9
227	erythronic acid	879	13752-84-6	6.9	0.1039	**0.0391**	5

a NIST match score value to reference database (scale: 0-999).

b Deviation between measured retention index (RI) and RI in reference database.

c Percent of change in means relative to control, positive value indicate higher in case.

**Figure 2:**
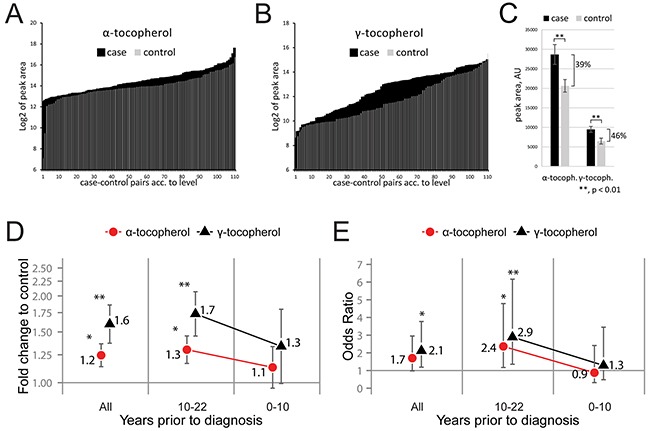
Elevated serum levels of both α- and γ-tocopherol in future glioblastoma cases **A–B.** Bar graph of GCxGC-TOFMS peak areas for α-tocopherol (A) and γ-tocopherol (B) in all measured sample, plotted in the order of peak area size. The plotted Log_2_ transformed raw data shows elevated levels of both α-tocopherol and γ-tocopherol in future glioblastoma cases. The graphs shows the obtained raw data, peak area of α- and γ tocopherol normalized to internal standards, without statistical interpretation. **C.** Average GCxGC-TOFMS peak area comparing α- and γ-tocopherol signal in cases to controls. A significant average increase of 39% for α-tocopherol and 46% for γ-tocopherol were measured in samples from cases. Error bars represent standard error of the mean. AU = arbitrary unit. **, p < 0.01. **D.** Fold change of α- and γ-tocopherol between case-control pairs in the whole study group (“All”) or subdivided according to years passed between sample collection and diagnosis (“0-10 years”, “10-22 years”). Error bars represent standard error of the mean. **E.** Odds ratios and 95% confidence intervals, calculated on log_2_ transformed and dichotomized metabolite levels by means of conditional logistic regression. Dichotomization was based on the median level among controls. A significant increase in fold change (D) and odds ratio (E) for the tocopherols were observed in future cases compared to controls. *, p < 0.05, **, p < 0.01.

The most prominent metabolite differences were high concentration of vitamin E variants α-tocopherol and γ-tocopherol in cases. Both α- and γ-tocopherol were considered significant when corrected for multiple comparisons using the Benjamini-Hochberg procedure [11] with an accepted false discovery rate below 0.2. The glioblastoma cases had an overall higher quantified peak area of both α-tocopherol and γ-tocopherol compared to controls (Figure 2A and 2B). In future glioblastoma cases, the relative levels of α- and γ-tocopherol showed a significant (p < 0.01) average increase of 39% and 46%, respectively (Figure 2C). Evaluating the tocopherol levels between the matched case-control pairs showed an average 1.6-fold increase (p = 0.002, Wilcoxon test) of γ-tocopherol and 1.2-fold increase (p = 0.015, Wilcoxon test) of α-tocopherol, in glioblastoma cases (Figure 2D). Subdividing the matched case-control pairs according to the time passed between serum sample collection and diagnosis showed a non-significant fold change of tocopherols in the 0-10-year subgroup, whereas the fold changes for both tocopherols in the 10-22-year subgroup were significantly elevated (Figure 2D). High tocopherol serum levels were associated with glioblastoma occurrence with an average odds ratio of 1.7 (95% CI: 1.0-3.0, p = 0.059) for α-tocopherol and 2.1 (95% CI: 1.2-3.8, p = 0.011) for γ-tocopherol (Figure 2E). Subdividing the glioblastoma cases stratified by time between blood collection and tumor diagnosis showed an increased odds ratio of 2.4 (95% CI: 1.2-4.8, p = 0.017) for α-tocopherol and 2.9 (95% CI: 1.4-6.2, p = 0.006) for γ-tocopherol, during the period 10-22 years before diagnosis. For all analyzed case-control pairs the calculated odds ratio per unit change in standard deviation was 1.49 and 1.56 for α- and γ-tocopherol, respectively. This calculation estimates that an increase in serum levels of tocopherols by one standard deviation increases the odds associated with future glioblastoma development with approximately 50%.

## DISCUSSION

In this study, we performed a large unbiased screen of the serum metabolome to identify small molecular compounds associated with future glioblastoma development. We found a novel association between high serum levels of vitamin E - i.e., α-tocopherol and γ-tocopherol - in future glioblastoma cases compared to their matched controls samples. In addition to the tocopherols, our screen identified elevated levels of erythritol, myo-inositol, erythronic acid, 2-keto-L-gluconic acid, cystine and hypoxantine, as well as reduced levels of xanthine in cases. This observation should be seen as exploratory and will need further validation in other prospective serum biobanks. Nevertheless, our screen points out several metabolites involved in the antioxidant metabolism and with potential involvement in the initiation of glioblastoma.

Our finding that high serum levels of vitamin E correlates with future glioblastoma development does not completely agree with findings from earlier dietary questionnaire studies [12–14]. However, dietary intake of vitamin E does not reflect the actual level of various tocopherol isoforms in serum and tissue due to post-adsorptive vitamin selective processes [15]. Vitamin E has been hypothesized to have an etiological role in several different tumor types. Initial studies indicated that dietary supplementation of a moderate dose of vitamin E could be associated with a reduced risk of prostate cancer [16]. However, large intervention trials with supplementation of vitamin E have been halted due to unfavorable results. In the SELECT trial, the investigators saw, after seven years of follow-up, a 17% increased risk of developing high-risk prostate cancer in men with dietary vitamin E supplementation [17]. The increased risk appeared three years after randomization, indicating a delayed-onset mechanism.

A recent metabolomics study investigating metabolic markers of adenocarcinoma, a type of non-small cell lung cancer, also found significantly elevated levels of both α- and γ-tocopherol in tumor tissue compared to nonmalignant tissue [18]. The underlying mechanisms resulting in high concentrations of circulating α- and γ-tocopherol and increased risk of glioblastoma needs further evaluation. Asthma and allergies are consistently associated with a reduced risk of glioma, indicating a role for inflammation in gliomagenesis [3–5]. Low levels of IgE have also been observed long before diagnosis and associated with glioma risk [19, 20]. Interestingly, tocopherols have anti-inflammatory effects through inhibition of cyclooxygenases (COX) and pro-inflammatory signaling such as NF-κB and STAT3/6 [21]. The long-chain intermediate metabolite of tocopherol, 13′-carboxychromanols, has an especially strong anti-inflammatory effects through direct COX1/2 inhibition [22]. The bioactivity among vitamin E forms may be entrenched in their distinct catabolism. It is likely that the majority of the long-chain carboxychromanols are generated through ω-hydroxylation of δ- and γ-tocopherol, since α-tocopherol is predominantly protected from degradation through αTTP binding.

A plausible mechanism for the observed association between high levels of tocopherols and glioma risk is that tocopherols suppress inflammation and thereby enhance the risk of developing brain tumors. As shown in animal studies, tocopherols reduce neuro-inflammatory effects in relation to epilepsy [23]. The anti-inflammatory effects of tocopherols, especially γ-tocopherol, have also been documented in animal studies investigating asthma and allergy [24, 25] and in several clinical studies [26]. Administration of tocopherols may also accelerate the progression of tumors. Interestingly, a recent study showed that supplementing the diet with antioxidants N-acetylcysteine or α-tocopherol increases tumor progression and reduces survival of RAS-induced mouse models of lung cancer [27]. In addition, this study shows that α-tocopherol supplementation reduces p53 expression in tumors and increases the proliferation rate of cancer cells [27]. Moreover, and further in line with our findings, the water soluble α-tocopherol analogue Trolox was recently shown to markedly increased the migration and invasive properties of human malignant melanoma cells [28]. Tocopherol intake may also affect DNA repair mechanisms [29].

Tocopherols are essential micronutrients involved in various oxidative stress-related processes. Because of their hydrophobic nature, tocopherols are transported in plasma lipoproteins, and the pathways involved in its cellular uptake are correlated to the lipoprotein metabolism. Transport in the circulation and cellular uptake depends on tocopherol binding proteins αTTP and SEC14L2. Cytoplasmic SEC14L2 is highly expressed in normal brain, breast, prostate, and liver tissues. Reduced expression of SEC14L2 has been reported in human breast cancer, indicating that SEC14L2 may serve as a tumor suppressor [30, 31]. In addition to its tocopherol binding mechanism, SEC14L2 binds phosphatidylinositol and is implicated in the regulation of the phosphatidylinositol 3-kinase/AKT signaling pathway, a pathway frequently disturbed in glioblastoma [32, 33]. For these reasons, binding of SEC14L2 to tocopherol and other interaction partners might be impaired in glioblastoma cases. However, this speculation warrants further investigation.

Susceptibility of brain tissues to oxidative damage has been studied intensively. Compared to other tissues, brain tissue has higher ROS production rates and lower ROS removal capacity [34]. ROS-related oxidation of DNA is one of the main causes of mutations, which can produce DNA damage that result in genomic instability and future development of carcinogenesis. In addition to vitamin E, our screen points out a latent biomarker including several molecules with antioxidant properties as well as oxidative stress generated end-products. Erythritol is a simple polyol with a chemical structure resembling that of mannitol, a well-known hydroxyl radical scavenger. Erythritol has excellent hydroxyl radical scavenger properties, but it is inert with respect to superoxide radicals [35]. The reaction of erythritol with hydroxyl radicals result in the formation of erythrose and erythrulose [35]. Erythrose can further be oxidized to form erythronic acid. Both erythrose and erythronic acid have been identified as products when glucose derived N-acetylglucosamine (GlcNAc) is oxidized by NaOCl, although only erythronic acid is formed by H_2_O_2_ oxidation [36]. Elevated levels of erythronic acid is a major hallmark of transaldolase deficiency. Elevated concentrations of ribitol, D-arabitol, and erythritol have also been identified in urine and plasma of transaldolase-deficient patients [37, 38]. Transaldolase is a key enzyme of the nonoxidative pentose phosphate pathway, providing ribos-5-phosphate for nucleic acid synthesis and NADPH for lipid biosynthesis. The transaldolase pathway also maintains glutathione in a reduced state to protect sulfhydyl groups and cellular integrity from oxygen radicals. In addition to elevated levels of erythronic acid and erythritol, our study also identified elevated levels of xylose, which is normally metabolized through the pentose phosphate pathway.

In future glioblastoma cases, our latent biomarker pointed out elevated levels of myo-inositol and cystine (Figure 1D). Both myo-inositol and cystine, the oxidized form of cysteine, have antioxidant properties and are involved in cellular redox homeostasis. Myo-inositol is one of the stereoisomers of inositol. Inositol occurs ubiquitously in cell membranes in conjugation with phospholipids. Inositol derivatives (e.g., variants of phosphatidylinositol) can act as pan protein kinase C (PKC) activators. A study in primary cultured enterocytes show doze-dependent activation of the antioxidant enzymes SOD, CAT, GPx and GST in inositol treated cells, which may be a result of PKC activation [39]. Elevated levels of myo-inositol have been detected in extracellular fluids from human brain tumors, compared to subcutaneous tissue fluids [40], and correlated to brain tumor grade (WHO II-IV) [41].

Reduced levels of xanthine was detected in serum from cases compared to matched control. Interestingly, the levels of hypoxantine was increased in cases whereas uric acid levels were unchanged. The observation indicates deregulated activity of the purine metabolism and the catalyzing enzyme xanthine oxidoreductase (XOR). XOR catalyzes the conversion of hypoxanthine to xanthine and xanthine to uric acid with concomitant reduction of either NAD^+^ or O_2_. XOR is a cytosolic protein with two forms - xanthine dehydrogenase and xanthine oxidase - and the potential to generate oxygen radical species (H_2_O_2_ and O_2_ ^−^) upon conversion [42]. Hypoxanthine oxidation is not per se a two-step reaction, as the xanthine intermediate is detached from the enzyme during hypoxanthine oxidation. Mutation of XOR could potentially shift the binding affinity for hypoxanthine towards xanthine, which would generate the observed metabolite pattern. Both loss-of-function, partial loss of activity, and gain-of-function point mutations of human XOR have been described [42, 43]. In addition, significantly higher xanthine oxidase levels have been reported in tumoral brain tissues [44].

Our study investigated pre-diagnosed serum samples, collected up to 22 years before glioblastoma diagnosis. We observed a time trend association between high α- and γ-tocopherol levels and glioblastoma risk throughout the investigated period. Unexpectedly, we found that the subgroup closest to diagnosis had a reduced fold change and odds ratio for the tocopherols, but this observation may be related to a delayed-onset mechanism. Due to the explorative nature of the study, our findings need to be scrutinized by future follow-up studies. Our multivariate analysis identified α- and γ-tocopherol as part of a significant metabolite pattern (a latent biomarker) which gives additional strength to the finding. The analysis highlights a correlation structure between mechanistically linked metabolites that together constitutes a biomarker more significant than any of the individual metabolites involved. To confirm a possible delayed onset mechanism would require analysis of pre-diagnostic samples from a longitudinal study with multiple serum samples collected over a long time. Our study, however, found a novel association of α- and γ-tocopherol with glioblastoma with a long latency time. This finding support recent findings in studies of other tumor sites and findings in prevention trials that concluded that high vitamin E levels may have negative health effects. In our case, the observed latent biomarker was detected several years before diagnosis.

## MATERIALS AND METHODS

### Study subjects and sample acquisition

Serum samples were obtained from the Janus Serum Bank a population-based serum biobank integrated into the Cancer Registry of Norway. The Janus serum samples were collected during the period from 1972-2004, and originate from approximately 318 000 persons in Norway who have participated in nationwide health studies, and from Red Cross blood donors in and around Oslo. As of December 31^st^ 2013, more than 69 000 donors have subsequently been diagnosed with cancer [45]. We included serum samples collected in 1986-1991 from incident cases diagnosed with glioblastoma between 0.5 and 22 years after sampling (Table 1). The Janus cohort was linked to the Cancer Registry of Norway to identify the study subjects. The Cancer Registry of Norway was founded in 1951 and is considered to capture close to 100% of new cancer cases yearly, and its completeness and quality has been evaluated [46]. The information comes from pathology laboratories, clinicians, the National Patients Discharge Registry and the Cause of Death Registry. All cases were primary glioblastomas (WHO grade IV). With the exception of one case of non-melanoma skin cancer, none of the cases had prior history of cancer. For each case, we randomly selected an undiseased control, matching on sex, age, blood collection year (+/− three months), sampling site and county. The majority of the samples came from people participating in health surveys with an average age of 44.2 years. All controls were free from cancer at the time of the cases diagnosis. The Janus Serum Bank sampling routine and long-term stability of serum components have been described previously [47]. The sampling routine was constant throughout the selected sample collection period. Samples were collected between 8 am and 6 pm from non-fasting donors, and blood was drawn with the donor in a supine position. Blood was collected in serum separator gel vials and allowed to clot at room temperature prior to transport and storage at the biobank at −25°C. The study group was homogeneous with respect to ethnicity resulting in a predominantly Caucasian cohort. All donors in the Janus Serum Bank have given informed broad consent for the use of their samples in cancer research. The study was approved by the Regional Committees for Medical and Health Research Ethics at the University of Oslo, Norway.

### Metabolite extraction from serum

The serum samples were divided into analytical batches, preserving case-control interrelation, and randomized prior to metabolite extraction. Frozen 50 μl aliquots of serum were thawed on ice at room temperature. Metabolite extraction was performed by addition of 450 μl methanol:water extraction mix (90:10 v/v), including internal standards (6.75 ng/μl), followed by rigorous agitation at 30 Hz for 2 minutes in a bead mill (Retsch, MM 400). The samples were incubated on ice for 2 hours and centrifuged at 18 600 × g (Eppendorf, 5417R) for 10 minutes at 4°C. After pre-clearing, 200 μl supernatant were transferred to GC vials and evaporated until dry in a speedvac (miVac, Quattro concentrator) and stored at −80°C. Sample derivatization was carried out prior to mass spectrometric analysis. Completely dried samples were methoxyaminated by the addition of 15 μl methoxyamine in pyridine (15 μg/μl), shaked for 10 minutes at room temperature and heated to 70°C for 60 minutes. The reaction was allowed to continue for 16 hours at room temperature. Trimethylsilylation was performed by addition of 15 μl MSTFA + 1% TMCS, and incubated for 1 hour at room temperature. Finally, 15 μl heptane, including methyl stearate (15 ng/μl), was added as an injection standard.

### Metabolite analysis by mass spectrometry

We analyzed the serum metabolites in randomized pairs within the analytical batches. The case and the corresponding control were consequently run in the same batch and directly adjacent to each other in the analytical run, thereby minimizing variability in platform performance across matched case-control pairs [48]. The samples were diluted 5-fold in heptane and analyzed with a Leco Pegasus 4D time-of-flight mass spectrometer (TOFMS) equipped with an Agilent 6890 comprehensive two-dimensional gas chromatograph (GCxGC) with quad-jet liquid nitrogen thermal modulator. Leco ChromaTOF software was used for instrument control and raw data acquisition. The column set used for the GCxGC separation was a primary low-polarity 30 m, 0.25 mm i.d. Rtx-5Sil MS column with 0.25 μm 5% diphenyl/95% dimethyl polysiloxane stationary phase (Restek, Bellefonte PA, USA), and a secondary mid-polar 2 m, 0.15 i.d. BPX-50 column with 0.15 μm 50% diphenyl/50% dimethylpolysiloxane film (SGE, Ringwood, Australia). Splitless injection of 1 μl sample was performed with an Agilent 7683B automated liquid sampler at an injection temperature of 270°C. The purge time was 60 seconds with a rate of 20 ml/min. Helium was used as carrier gas with a flow rate of 1 ml/min. The primary GC oven temperature was held constant at 60°C for 2 minutes and then ramped at 4°C/minute to 300°C, where it was held constant for 5 minutes. The secondary GC oven maintained the same temperature program with an offset of +40°C compared to the primary oven. The modulation period was set to 5 seconds with a 1.0 second hot pulse and a 1.5 second cooling time between the stages. The transfer line temperature between the gas chromatograph and mass spectrometer was set to 325°C. Electron impact ionization at 70 eV was employed with an ion source temperature of 250°C. Mass spectra were collected in the mass range of m/z 50 to 600 at 100 Hz and 1900V detector voltage. A series of n-alkanes (C8-C40) were used as external retention index (RI) standards. As an additional quality control measure of analytical performance across and within samples batches, we analyzed a pooled serum quality control reference sample at the beginning and end of each analytical batch, as well as following every 20^th^ study sample.

### Metabolite identification and quantification

Acquired raw data was exported to MATLAB (Mathworks, Natick, MA) in NetCDF format and processed using the hierarchical multivariate curve resolution (H-MCR) script, developed in house [49] and further adapted for two dimensional GC data. The H-MCR procedure generates chromatographic profiles for each compound in each sample with a corresponding common spectral profile. We used the integrated area under the resolved chromatographic profile for quantification. The identity of the resolved peaks were determined by comparing mass spectra and retention indices with data in mass spectral libraries, including the Swedish Metabolomics Centre in-house library, the Golm metabolome database (http://gmd.mpimp-golm.mpg.de/) and the NIST Standard Reference Database, using NIST MS search 2.0. Compounds with a “spectral match score” above 700 and RI deviation no larger than 20 units from the reference value, were selected and manually evaluated for identification. For identification with high confidence, all major fragment ions in the library hit should be present in the resolved spectra with correct spectral intensity profile. Both identified peaks and unidentified peaks were included in the multivariate analysis.

### Statistical modeling and analysis

Multivariate analysis was applied to the data in order to extract patterns of metabolites, latent biomarkers, associated with future glioblastoma diagnosis. Effect projections by means of orthogonal partial least squares (OPLS-EP), a multivariate projection approach developed for dependent or matched samples, was used to focus on the changes in metabolite composition associated by common difference between glioblastoma case and control over all matched sample pairs [48]. Variables with a variable influence on projection >1 [50], were used for modelling. The model estimate Yhat (Yhat=1, model target) were used to visualize the magnitude of the effect for each matched pair, while the model variable weights (loadings, p1) was used to highlight the metabolites contributing most to the projected effect. The predictive ability of the model (Q^2^) was decided by full (seven-fold) cross validation yielding a value between -∞ and 1, where 1 refers to a perfect prediction. The model significance was calculated using the cross-validated effect score yielding a p-value for the common case-control effect on the metabolite profile [51]. The p-value for the extracted latent variables were calculated using paired two-tailed Student's t-test for the difference between case and control, described by the predictive component of the OPLS-EP models. All multivariate statistical analyses were performed in the SIMCA 13.0.3 software (Umetrics AB, Sweden). Univariate statistical analysis of the identified compounds was done on the log_2_ transformed peak area data, as a second step in the process of sequestering important findings from the metabolic profiles. Case-control pairing prior to analysis enabled the use of paired two-tailed Student's t-test for further verification of the findings in the multivariate modeling and to provide significance testing of the identified metabolites on an individual basis. Average fold change was calculated on the log_2_ transformed case-control fold change values. The geometric average was back-transformed [2^x^] to enable visualization on a normal fold change scale, where 1 indicates no change. Two pairs with a >50-fold increase in cases compared to controls were excluded from the average fold change calculations. A non-parametric Wilcoxon signed-rank test was used for statistical testing of the not normally distributed fold-change calculations. We used conditional logistic regression on the log-transformed concentration data to assess the effect size of differences in metabolite levels between cases and controls. Odds ratios (ORs) and 95% confidence intervals (CIs) were calculated both for comparing samples with high metabolite levels to samples with low metabolite levels, where the dichotomization was based on the median level among controls, and per standard deviation increase in log-transformed metabolite levels.

### Special reagents

All chemicals were of analytical grade. The isotopically labeled internal standards (IS) [1,2,3-13C3]-myristic acid and [2H6]-salicylic acid were purchased from Cambridge Isotope Laboratories (Andover, MA, USA) and Icon (Summit, NJ, USA) respectively. The stock solutions for IS were prepared in 0.5 μg/μl concentrations in methanol prior to metabolite extraction. Silylation grade pyridine and N-Methyl-N-trimethylsilyltrifluoroacetamide (MSTFA) with 1% trimethylchlorosilane (TMCS) were purchased from Pierce Chemical Co (Rockford, IL, USA).

## SUPPLEMENTARY TABLE




