# Supplementary material for: Metabolomic screening of pre-diagnostic serum samples identifies association between α- and γ-tocopherols and glioblastoma risk
Source: Oncotarget. 2016 May 9;7(24):37043–53. doi: 10.18632/oncotarget.9242 (PMC5095057; doi:10.18632/oncotarget.9242)
Supplement: Supplementary file 1 [file oncotarget-07-37043-s001.pdf]

Metabolomic screening of pre-diagnostic serum samples identifies association between α- and γ-tocopherols and glioblastoma risk

SUPPLEMENTARY TABLE

Supplementary Table S1: Supplementary table of all identified serum metabolites in the matched case-control pairs, with univariate statistical interpretation.

See Supplementary File 1
