# Supplementary material for: Metabolomic screening of pre-diagnostic serum samples identifies association between α- and γ-tocopherols and glioblastoma risk
Source: Oncotarget. 2016 May 9;7(24):37043–53. doi: 10.18632/oncotarget.9242 (PMC5095057; doi:10.18632/oncotarget.9242)
Supplement: Supplementary file 2 [file oncotarget-07-37043-s002.docx]

| **Supplementary table 1** | | | |  |  |  |  |  |  | **p-value** | **p-value** |  | **mean peak area (AU)** |  |
| --- | --- | --- | --- | --- | --- | --- | --- | --- | --- | --- | --- | --- | --- | --- |
| **Primary ID** | **RT 1D (sec)** | **RT 2D (sec)** | **Identification** | **Match^a^** | **R.Match** | **CAS#** | **RI (DB)** | **RI^b^** | **ΔRI** | **(t-test)** | **(paired t-test)** | **GBM case** | **control** | **change (%)^d^** |
| 12 | 535 | 4,45 | N-Methylmaleimide | 915 | 924 | 930-88-1 | 925 | 946.1 | 21.1 | 0.7520 | 0.6061 | 22470.6 | 22021.7 | 2 |
| 14 | 570 | 2,42 | Carbodiimide (2TMS) | 903 | 952 | 1000-70-0 | 953 | 962.2 | 9.2 | 0.8369 | 0.7021 | 4626931.5 | 4582946.0 | 1 |
| 19 | 595 | 2,6 | Pentanoic acid, trimethylsilyl ester | 922 | 922 | 26429-16-3 | 974 | 974.4 | 0.4 | 0.1492 | 0.1264 | 201192.3 | 246358.6 | -18 |
| 24 | 640 | 2,29 | 3,6-Dioxa-2,7-disilaoctane, 2,2,4,7,7-pentamethyl- | 941 | 941 | 17887-27-3 | 1013 | 996.2 | 16.8 | 0.8324 | 0.6794 | 7163727.1 | 7228553.1 | -1 |
| 28 | 685 | 2,04 | 1,1,1-trimethyl-N-propyl-N-(trimethylsilyl)-silanamine | 777 | 790 | 7331-84-2 | 1031 | 1018.1 | 12.9 | 0.4281 | 0.2083 | 388170.1 | 376204.7 | 3 |
| 29 | 680 | 2,38 | 2-Heptanol, trimethylsilyl ether | 910 | 911 | 53690-75-8 | 1009 | 1015.8 | 6.8 | 0.5612 | 0.2677 | 646780.6 | 640527.0 | 1 |
| 31 | 700 | 2,6 | 4-Decene, 7-methyl-, (E)- | 816 | 823 | 62338-48-1 | 1059 | 1025.7 | 33.3 | 0.7074 | 0.3736 | 418451.5 | 414347.3 | 1 |
| 32 | 690 | 4,55 | 3,6-Heptanedione | 836 | 894 | 1703-51-1 | 1041 | 1021.7 | 19.3 | 0.7684 | 0.1875 | 25756.2 | 25519.1 | 1 |
| 36 | 715 | 3,23 | Pyridine, 2-hydroxy- (1TMS) | 929 | 943 | 142-08-5 | 1031 | 1033.3 | 2.3 | 0.7245 | 0.5729 | 114112.4 | 113376.5 | 1 |
| 41 | 760 | 2,62 | LACTIC ACID, O,O-TMS | 962 | 968 | 17596-96-2 | 1067 | 1054.9 | 12.1 | 0.1807 | 0.0842 | 20770332.8 | 19397393.2 | 7 |
| 42 | 790 | 2,69 | Hexanoic acid, trimethylsilyl ester | 947 | 959 | 14246-15-2 | 1071 | 1069.6 | 1.4 | 0.2864 | 0.2322 | 665840.0 | 788693.2 | -16 |
| 46 | 850 | 2,6 | ALANINE-N,O-TMS | 957 | 957 | 56-41-7 | 1113 | 1098.8 | 14.2 | 0.6001 | 0.4578 | 2438918.5 | 2256335.7 | 8 |
| 50 | 865 | 3,32 | Nonanal | 885 | 896 | 124-19-6 | 1104 | 1106.3 | 2.3 | 0.3232 | 0.2184 | 8209.0 | 6894.0 | 19 |
| 53 | 875 | 2,28 | Hydroxylamine (3TMS) | 882 | 898 | 21023-20-1 | 1101 | 1110.6 | 9.6 | 0.5407 | 0.4215 | 53368.1 | 54539.2 | -2 |
| 54 | 890 | 2,63 | GLYCINE,N,O-TMS | 929 | 929 | 7364-42-3 | 1105 | 1117.9 | 12.9 | 0.6526 | 0.2584 | 62533.9 | 58025.9 | 8 |
| 55 | 900 | 2,51 | Butanoic acid, 2-[(trimethylsilyl)oxy]-, trimethylsilyl ester | 902 | 902 | 55133-93-2 | 1130 | 1122.6 | 7.4 | 0.6722 | 0.6662 | 717871.6 | 693215.5 | 4 |
| 56 | 905 | 2,53 | 1) Oxalic acid (2TMS) | 718 | 803 | 18294-04-7 | 1134 | 1125 | 9 | 0.7015 | 0.5403 | 139468.9 | 140810.8 | -1 |
| 60 | 930 | 2,93 | 2) Oxalic acid (2TMS) | 722 | 757 | 18294-04-7 | 1134 | 1137.1 | 3.1 | 0.5377 | 0.0967 | 9642816.0 | 9849945.1 | -2 |
| 69 | 955 | 3,3 | Silane, trimethyl(3-methylphenoxy)- | 909 | 913 | 17902-31-7 | 1134 | 1149.2 | 15.2 | 1.0000 | 1.0000 | 328732.5 | 333428.6 | -1 |
| 77 | 970 | 3,41 | Sulfuric acid (2TMS) | 940 | 943 | 7664-93-9 | 1162 | 1156.4 | 5.6 | 0.8662 | 0.7271 | 66576.0 | 72643.8 | -8 |
| 78 | 980 | 1,99 | Butanoic acid, 3-methyl-2-[(trimethylsilyl)oxy]-, trimethylsilyl ester | 879 | 886 | 55124-92-0 | 1172 | 1160.5 | 11.5 | 0.8861 | 0.8688 | 46087.7 | 49096.3 | -6 |
| 79 | 975 | 2,66 | 3-HYDROXYBUTYRIC ACID-2TMS | 948 | 949 | 300-85-6 | 1168 | 1158.4 | 9.6 | 0.4170 | 0.3913 | 328348.1 | 495305.9 | -34 |
| 87 | 1005 | 3,39 | Phosphoric acid, bis(trimethylsilyl)monomethyl ester | 832 | 903 | 18291-81-1 | 1167 | 1173 | 6 | 0.3195 | 0.0948 | 174670.6 | 164730.5 | 6 |
| 92 | 1045 | 2,61 | 3-Dodecene, (Z)- | 882 | 949 | 7239-23-8 | 1195 | 1191.7 | 3.3 | 0.9534 | 0.9192 | 60140.1 | 60020.3 | 0 |
| 100 | 1065 | 2,55 | n-Dodecane | 929 | 929 | 112-40-3 | 1200 | 1201.2 | 1.2 | 0.8404 | 0.7395 | 50596.7 | 49758.9 | 2 |
| 101 | 1075 | 2,52 | 3-Hydroxy-3-methyl-Butanoic acid (2TMS) | 884 | 886 | 55124-90-8 | 1210 | 1206.1 | 3.9 | 0.2028 | 0.2054 | 52631.2 | 50187.3 | 5 |
| 103 | 1090 | 2,6 | VALINE,N,O-TMS | 909 | 910 | 7364-44-5 | 1234 | 1213.5 | 20.5 | 0.6953 | 0.5774 | 2672716.6 | 2706597.4 | -1 |
| 108 | 1120 | 2,46 | 1) ETHANOLAMINE,N,N,O-TMS-like | 826 | 834 | 5630-81-9 | 1265 | 1228 | 37 | 0.8111 | 0.4388 | 2376657.3 | 2368087.0 | 0 |
| 113 | 1145 | 3,53 | UREA-2TMS | 932 | 932 | 18297-63-7 | 1243 | 1240.7 | 2.3 | 0.3746 | 0.3054 | 40201482.9 | 39052705.8 | 3 |
| 116 | 1155 | 3,65 | Benzoic acid, (1TMS) | 881 | 921 | 65357 | 1251 | 1245.7 | 5.3 | 0.9571 | 0.9368 | 190389.1 | 188499.6 | 1 |
| 120 | 1180 | 2,58 | 4-Trimethylsiloxy(trimethylsilyl)valerate | 880 | 889 | 72101-10-1 | 1265 | 1257.4 | 7.6 | 0.4235 | 0.4435 | 14072.2 | 16555.3 | -15 |
| 122 | 1175 | 2,84 | Serine, bis(trimethylsilyl)- | 890 | 895 | 70125-39-2 | 1252 | 1255 | 3 | 0.8495 | 0.7550 | 334512.1 | 332123.2 | 1 |
| 125 | 1180 | 4,09 | N-ACETYL-L-ALANINE-TMS | 723 | 862 | 1115-69-1 | 1257 | 1258.1 | 1.1 | 0.1414 | **0.0314** | 10395.9 | 9900.4 | 5 |
| 126 | 1195 | 2,3 | 2) ETHANOLAMINE,N,N,O-TMS | 927 | 927 | 5630-81-9 | 1265 | 1264.5 | 0.5 | 0.3683 | 0.2228 | 258599.7 | 250360.0 | 3 |
| 133 | 1210 | 2,24 | Glycerol (3TMS) | 911 | 917 | 1785220 | 1284 | 1271.8 | 12.2 | 0.7538 | 0.7081 | 11975752.6 | 11833057.1 | 1 |
| 136 | 1205 | 2,56 | LEUCINE, N,O-TMS | 915 | 915 | 7364-46-7 | 1272 | 1269.5 | 2.5 | 0.9924 | 0.9877 | 977785.9 | 961434.3 | 2 |
| 137 | 1205 | 2,82 | Phosphoric acid (3TMS) | 881 | 889 | 10497-05-9 | 1285 | 1269.7 | 15.3 | 0.1867 | 0.0578 | 10333484.6 | 10034823.5 | 3 |
| 144 | 1225 | 4,23 | Glycine, N-acetyl-, trimethylsilyl ester | 947 | 949 | 25436-19-5 | 1253 | 1280.1 | 27.1 | 0.4549 | 0.3847 | 21861.0 | 20084.3 | 9 |
| 146 | 1255 | 2,94 | 1) L-Proline (2TMS) | 894 | 907 | 7364-47-8 | 1301 | 1294.1 | 6.9 | 0.3140 | 0.2250 | 3450629.4 | 3688159.4 | -6 |
| 149 | 1255 | 4,04 | Benzeneacetic acid, trimethylsilyl ester | 865 | 895 | 2078-18-4 | 1303 | 1294.7 | 8.3 | 0.5791 | 0.4560 | 19886.8 | 19082.9 | 4 |
| 154 | 1275 | 2,58 | GLYCINE,N,N,O-TMS | 937 | 937 | 5630-82-0 | 1304 | 1303.8 | 0.2 | 0.7925 | 0.7799 | 5257119.6 | 5278366.2 | 0 |
| 158 | 1320 | 2,61 | Glyceric acid (3TMS) | 882 | 898 | 38191-87-6 | 1332 | 1326.3 | 5.7 | 0.4002 | 0.3373 | 503810.1 | 482401.1 | 4 |
| 165 | 1390 | 0,24 | 2(3H)-Furanone, dihydro-5-pentyl- | 899 | 918 | 104-61-0 | 1325 | 1360.1 | 35.1 | 0.6427 | 0.2720 | 3379.2 | 3252.0 | 4 |
| 166 | 1380 | 2,59 | L-Serine (3TMS) | 893 | 893 | 7364-48-9 | 1369 | 1356.3 | 12.7 | 0.5150 | 0.4613 | 2040816.9 | 2067005.0 | -1 |
| 167 | 1385 | 2,86 | Nonanoic acid (1TMS) | 882 | 937 | 82326-11-2 | 1358 | 1358.9 | 0.9 | 0.9833 | 0.9741 | 388624.5 | 389383.1 | 0 |
| 172 | 1430 | 2,58 | L-Threonine (3TMS) | 897 | 923 | 7537-02-2 | 1394 | 1381.3 | 12.7 | 0.7261 | 0.7139 | 3674159.8 | 3682714.9 | 0 |
| 178 | 1465 | 2,64 | Tetradecane | 862 | 921 | 629-59-4 | 1400 | 1398.8 | 1.2 | 0.6606 | 0.3639 | 22893.9 | 22321.1 | 3 |
| 181 | 1460 | 3,24 | Alanine {BP} (3TMS) | 889 | 911 | 56-41-7 | 1409 | 1396.6 | 12.4 | 0.6110 | 0.3320 | 42498.5 | 38423.9 | 11 |
| 183 | 1510 | 2,53 | BETA-ALANINE, N,N,O-TMS | 951 | 951 | 55255-77-1 | 1423 | 1423 | 0 | 0.9540 | 0.9514 | 78114.3 | 77133.1 | 1 |
| 184 | 1505 | 3,29 | L-Aspartic acid, bis(trimethylsilyl) ester | 924 | 924 | 5269-42-1 | 1412 | 1420.7 | 8.7 | 0.6939 | 0.5164 | 117608.3 | 119297.4 | -1 |
| 185 | 1520 | 2,53 | 3,4-Dihydroxybutyric acid | 837 | 842 | 55191-53-2 | 1457 | 1428.4 | 28.6 | 0.0864 | 0.0709 | 62828.9 | 56094.5 | 12 |
| 193 | 1570 | 2,62 | Decanoic acid, trimethylsilyl ester | 873 | 877 | 55494-15-0 | 1455 | 1455.5 | 0.5 | 0.9056 | 0.8954 | 128385.8 | 131727.6 | -3 |
| 194 | 1565 | 3,42 | Ornithine-1,5-lactam (2TMS) | 796 | 928 | 32565-12-1 | 1454 | 1453.2 | 0.8 | 0.8145 | 0.6540 | 72580.3 | 72644.7 | 0 |
| 200 | 1625 | 2,8 | MALIC ACID-3TMS | 903 | 903 | 65143-63-7 | 1487 | 1485.3 | 1.7 | 0.4334 | 0.4069 | 110078.6 | 104057.5 | 6 |
| 203 | 1645 | 0,37 | 1) 2-Pyrrolidone-5-carboxylic acid, trimethylsilyl ester | 767 | 800 | 4042-36-8 | 1511 | 1494.8 | 16.2 | 0.9924 | 0.9887 | 108269.0 | 107791.3 | 0 |
| 206 | 1645 | 3,61 | Asparagine [-H2O] (2TMS) | 734 | 961 | 70-47-3 | 1501 | 1496.5 | 4.5 | 0.8245 | 0.7672 | 12068.4 | 11335.6 | 6 |
| 207 | 1660 | 3,14 | ADIPIC ACID-2TMS | 922 | 922 | 18105-31-2 | 1498 | 1504.5 | 6.5 | 0.9034 | 0.8362 | 424351.9 | 424084.4 | 0 |
| 210 | 1660 | 3,67 | SALICYLIC ACID-2TMS, * | 721 | 880 | 69-72-7 | 1505 | 1504.8 | 0.2 | 0.6354 | 0.5541 | 299536.0 | 199559.7 | **50** |
| 211 | 1665 | 2,17 | ERYTHRITOL-(4TMS) | 936 | 936 | 149-32-6 | 1505 | 1506.8 | 1.8 | 0.0506 | **0.0221** | 164863.1 | 151972.7 | 8 |
| 212 | 1685 | 0,05 | 2) 2-Pyrrolidone-5-carboxylic acid, trimethylsilyl ester | 810 | 834 | 4042-36-8 | 1511 | 1516.7 | 5.7 | 0.3626 | 0.0763 | 38110.2 | 36389.8 | 5 |
| 213 | 1685 | 2,77 | ASPARTIC ACID,N,N,O-TMS | 903 | 908 | 56-84-8 | 1516 | 1518.2 | 2.2 | 0.3996 | 0.2986 | 964654.8 | 980555.6 | -2 |
| 214 | 1680 | 2,8 | METHIONINE, N,O-TMS | 885 | 885 | 63-68-3 | 1515 | 1515.4 | 0.4 | 0.6971 | 0.6480 | 10968.2 | 10522.6 | 4 |
| 216 | 1680 | 3,98 | PYROGLUTAMIC ACID,N,O-TMS | 901 | 904 | 98-79-3 | 1514 | 1516.1 | 2.1 | 0.1510 | 0.1115 | 5025593.0 | 4887450.2 | 3 |
| 217 | 1695 | 2,67 | 4-HYDROXYPROLINE,N,O,O-TMS | 911 | 912 | 51-35-4 | 1522 | 1523.7 | 1.7 | 0.6825 | 0.6495 | 213595.0 | 209861.1 | 2 |
| 218 | 1705 | 3,47 | N-Acetylglutamic acid (2TMS) | 733 | 890 | 1188-37-0 | 1532 | 1529.7 | 2.3 | 0.7962 | 0.7450 | 335321.3 | 340278.3 | -1 |
| 224 | 1715 | 2,91 | Ditertbutylphenol | 784 | 892 | 88-26-6 | 1542 | 1535 | 7 | 0.4977 | 0.1626 | 25670.7 | 25319.9 | 1 |
| 227 | 1725 | 2,25 | Erythronic acid (4TMS) | 879 | 898 | 13752-84-6 | 1547 | 1540.1 | 6.9 | 0.1039 | **0.0391** | 281364.8 | 269064.4 | 5 |
| 229 | 1730 | 4,38 | Phenylalanine (1TMS) | 937 | 937 | 2899-42-5 | 1556 | 1544.1 | 11.9 | 0.9331 | 0.8625 | 221088.5 | 220459.7 | 0 |
| 230 | 1740 | 3,17 | CREATININE-3TMS | 926 | 926 | 60-27-5 | 1550 | 1549 | 1 | 0.9940 | 0.9930 | 522971.5 | 532649.2 | -2 |
| 234 | 1755 | 2,5 | Threonic acid (4TMS) | 900 | 906 | 3909-12-04 | 1567 | 1556.9 | 10.1 | 0.2453 | 0.2025 | 519101.3 | 486385.7 | 7 |
| 236 | 1775 | 2,63 | Silane, (dodecyloxy)trimethyl- | 898 | 899 | 6221-88-1 | 1575 | 1568.1 | 6.9 | 0.9007 | 0.8180 | 113791.9 | 115363.2 | -1 |
| 237 | 1780 | 2,64 | Glutaric acid, 2-hydroxy- (3TMS) | 723 | 924 | 55530-62-6 | 1569 | 1570.9 | 1.9 | 0.8883 | 0.8261 | 141645.1 | 141738.5 | 0 |
| 241 | 1790 | 3,73 | 2) Proline (2TMS) | 899 | 908 | 7364-47-8 | 1593 | 1577.1 | 15.9 | 0.8583 | 0.6759 | 194480.4 | 191573.6 | 2 |
| 245 | 1810 | 3,36 | Propanoic acid, 2-methyl-, 1-(1,1-dimethylethyl)-2-methyl-1,3-propanediyl ester | 937 | 942 | 74381-40-1 | 1591 | 1588 | 3 | 0.6931 | 0.3594 | 231992.4 | 211907.1 | 9 |
| 250 | 1830 | 2,96 | HEPTANEDIOIC ACID-2TMS | 947 | 947 | 111-16-0 | 1600 | 1598.9 | 1.1 | 0.9668 | 0.9216 | 547373.3 | 546321.4 | 0 |
| 256 | 1850 | 2,55 | CITRULLINE-3TMS_1 (Ornthine) | 936 | 936 | 24595-70-8 | 1608 | 1610.6 | 2.6 | 0.8819 | 0.7824 | 123767.5 | 120480.4 | 3 |
| 259 | 1845 | 3,43 | Benzoic acid, 2-[(trimethylsilyl)amino]-, trimethylsilyl ester | 738 | 844 | 18406-07-0 | 1600 | 1608.1 | 8.1 | 0.6229 | 0.6219 | 5770.4 | 6233.9 | -7 |
| 262 | 1860 | 2,91 | GLUTAMIC ACID,N,O,O-TMS | 799 | 845 | 56-86-0 | 1613 | 1616.9 | 3.9 | 0.9000 | 0.8569 | 4038996.9 | 4027324.9 | 0 |
| 264 | 1865 | 3,39 | PHENYLALANINE,N,O-TMS | 926 | 928 | 63-91-2 | 1622 | 1620.2 | 1.8 | 0.8406 | 0.7944 | 1486815.3 | 1493280.3 | 0 |
| 271 | 1890 | 3,37 | 4-HYDROXYPHENYLACETIC ACID-2TMS | 921 | 921 | 156-38-7 | 1637 | 1635.4 | 1.6 | 0.9676 | 0.9689 | 13782.3 | 13509.6 | 2 |
| 277 | 1915 | 2,92 | Dodecanoic acid (1TMS) | 925 | 925 | 55520-95-1 | 1654 | 1650.3 | 3.7 | 0.8727 | 0.8686 | 196089.5 | 202942.1 | -3 |
| 284 | 1925 | 1,63 | Ribose, D- (1MEOX) (4TMS) | 799 | 840 | 56196-08-8 | 1666 | 1655.5 | 10.5 | 0.6663 | 0.5389 | 49011.8 | 50000.6 | -2 |
| 285 | 1930 | 2,34 | XYLOSE-MEOX 4TMS_1 | 915 | 915 | 58-86-6 | 1651 | 1659 | 8 | 0.0566 | **0.0386** | 73533.9 | 67288.7 | 9 |
| 286 | 1925 | 2,48 | 3,4,5-Trihydroxypentanoic acid, tetrakis(trimethylsilyl)- | 883 | 888 | 29625-75-0 | 1652 | 1656 | 4 | 0.9520 | 0.9457 | 56547.3 | 57794.9 | -2 |
| 292 | 1940 | 2,69 | L-Asparagine (3TMS) | 930 | 930 | 55649-62-2 | 1664 | 1665.3 | 1.3 | 0.7603 | 0.5761 | 79108.7 | 72370.7 | 9 |
| 293 | 1935 | 3,01 | TAURINE-3TMS | 922 | 922 | 107-35-7 | 1664 | 1662.4 | 1.6 | 0.4691 | 0.2264 | 852275.0 | 876654.3 | -3 |
| 308 | 1990 | 3,11 | Octanedioic acid, bis(trimethylsilyl) ester | 894 | 906 | 43199-48 | 1689 | 1695.8 | 6.8 | 0.8761 | 0.7667 | 629197.6 | 632961.6 | -1 |
| 313 | 2005 | 2,03 | XYLITOL-5TMS | 918 | 918 | 14199-72-5 | 1700 | 1704.4 | 4.4 | 0.9984 | 0.9983 | 25279.2 | 22106.4 | 14 |
| 317 | 2025 | 2,13 | RIBITOL-5TMS | 907 | 907 | 32381-53-6 | 1721 | 1717 | 4 | 0.1323 | 0.0818 | 135735.6 | 129391.6 | 5 |
| 318 | 2030 | 2,56 | GLYCEROL-2-PHOSPHATE-4TMS | 915 | 915 | 15804-51-0 | 1705 | 1720.4 | 15.4 | 0.2721 | 0.1409 | 47806.5 | 46044.5 | 4 |
| 321 | 2035 | 3,37 | Tetradecanoic acid methyl ester, n- | 930 | 931 | 124-10-7 | 1722 | 1724 | 2 | 0.6965 | 0.6738 | 18232.7 | 19095.1 | -5 |
| 329 | 2095 | 2,15 | 1) 2-Keto-L-gluconic acid (5TMS) | 871 | 925 | 29123-55-5 | 1774 | 1760.7 | 13.3 | **0.0109** | **0.0070** | 18563.6 | 16930.0 | 10 |
| 330 | 2090 | 2,47 | GLYCEROL-3-PHOSPHATE-4TMS | 911 | 911 | 55073-41-1 | 1741 | 1757.8 | 16.8 | 0.5472 | 0.4380 | 1281041.0 | 1261641.5 | 2 |
| 334 | 2110 | 2,98 | L-Glutamine (3TMS) | 894 | 896 | 56145-13-2 | 1765 | 1770.6 | 5.6 | 0.8997 | 0.8749 | 157875.6 | 151818.6 | 4 |
| 337 | 2120 | 2,44 | 2) 2-Keto-L-gluconic acid (5TMS) | 857 | 927 | 29123-55-5 | 1774 | 1776.5 | 2.5 | 0.9741 | 0.9721 | 65787.8 | 65544.0 | 0 |
| 347 | 2150 | 3,32 | Azelaic acid, bis(trimethylsilyl) ester | 930 | 930 | 17906-08-0 | 1787 | 1795.8 | 8.8 | 0.8509 | 0.6134 | 7064036.1 | 7210424.0 | -2 |
| 351 | 2155 | 4,33 | HYPOXANTHINE-(2TMS) | 932 | 932 | 17962-89-9 | 1801 | 1799.6 | 1.4 | 0.4316 | 0.3903 | 82068.6 | 70324.4 | 17 |
| 353 | 2180 | 2,64 | ORNITHINE,N,N,N,O-TMS_2 | 912 | 912 | 55556-70-2 | 1814 | 1814.6 | 0.6 | 0.7674 | 0.6760 | 1029456.8 | 1031807.1 | 0 |
| 354 | 2180 | 2,86 | CITRIC ACID-4TMS | 914 | 914 | 77-92-9 | 1814 | 1814.7 | 0.7 | 0.6648 | 0.6250 | 1202371.3 | 1190909.1 | 1 |
| 361 | 2195 | 3,44 | Pentadecanoic acid methyl ester, n- | 897 | 897 | 7132-64-1 | 1822 | 1824.8 | 2.8 | 0.2017 | 0.1216 | 11817.5 | 11337.6 | 4 |
| 362 | 2210 | 1,84 | D-Pinitol (5TMS), * | 835 | 840 | 10284-63-6 | 1832 | 1833.4 | 1.4 | 0.5055 | 0.5070 | 2603.5 | 2976.3 | -13 |
| 363 | 2210 | 2,75 | MYRISTOLEIC ACID-TMS | 950 | 950 | 544-64-9 | 1834 | 1834 | 0 | 0.8147 | 0.8120 | 53307.2 | 56306.7 | -5 |
| 367 | 2220 | 0,48 | HIPPURIC ACID-TMS | 928 | 946 | 2078-24-2 | 1841 | 1839 | 2 | 0.6569 | 0.6544 | 106557.9 | 101604.9 | 5 |
| 369 | 2230 | 2,77 | 1,5-ANHYDRO-D-GLUCITOL-4TMS | 911 | 911 | 154-58-5 | 1843 | 1846.9 | 3.9 | 0.6589 | 0.6521 | 4836521.1 | 4921565.2 | -2 |
| 374 | 2240 | 2,34 | 1) Ketohexose (sorbose) MEOX 5TMS | 883 | 883 | 3615-56-3 | 1863 | 1853.1 | 9.9 | 0.9860 | 0.9859 | 30948.4 | 32512.1 | -5 |
| 375 | 2240 | 2,46 | D(-)-Quinic acid (5TMS) | 847 | 847 | 77-95-2 | 1862 | 1853.2 | 8.8 | 0.9054 | 0.9049 | 75527.2 | 79146.2 | -5 |
| 376 | 2255 | 0,24 | 1,2-Benzenedicarboxylic acid, bis(2-methylpropyl) ester | 906 | 906 | 84-69-5 | 1863 | 1861.4 | 1.6 | 0.6641 | 0.4986 | 12835.5 | 12601.2 | 2 |
| 377 | 2260 | 2,45 | 2) Ketohexose (sorbose) MEOX 5TMS | 925 | 925 | 3615-56-3 | 1863 | 1866.1 | 3.1 | 0.3036 | 0.2815 | 1641299.6 | 1417773.6 | 16 |
| 385 | 2275 | 2,47 | Ketohexose (fructose) MEOX 5TMS | 892 | 892 | 7660-25-5 | 1875 | 1875.8 | 0.8 | 0.3784 | 0.3534 | 963042.1 | 885379.0 | 9 |
| 387 | 2280 | 4,04 | Tyrosine (2TMS) | 960 | 960 | 7536-83-6 | 1887 | 1880 | 7 | 0.6610 | 0.4121 | 53266.3 | 55089.9 | -3 |
| 388 | 2300 | 2,45 | 1) Aldohexoses (glucose) MEOX 5TMS | 950 | 950 | 34152-44-8 | 1889 | 1891.9 | 2.9 | 0.7661 | 0.5530 | 8232107.4 | 8183042.4 | 1 |
| 389 | 2300 | 2,51 | 2) Aldohexoses (glucose) MEOX 5TMS | 932 | 935 | 34152-44-8 | 1889 | 1891.9 | 2.9 | 0.9518 | 0.9340 | 9709956.3 | 9632703.6 | 1 |
| 390 | 2300 | 2,51 | 3) Aldohexoses (glucose) MEOX 5TMS | 944 | 946 | 34152-44-8 | 1889 | 1891.9 | 2.9 | 0.8429 | 0.7776 | 15374843.5 | 15481053.4 | -1 |
| 392 | 2325 | 1,05 | THEOPHYLLINE-TMS, * | 949 | 949 | 62374-32-7 | 1912 | 1907.6 | 4.4 | 0.8095 | 0.7938 | 10042.7 | 11524.3 | -13 |
| 393 | 2330 | 2,47 | Aldohexose, galactose MEOX 5TMS | 948 | 948 | 59-23-4 | 1906 | 1912 | 6 | 0.4229 | 0.2288 | 10793175.2 | 10911375.2 | -1 |
| 396 | 2340 | 2,67 | LYSINE,N,N,N,O-TMS_2 | 914 | 914 | 55429-07-7 | 1915 | 1919.1 | 4.1 | 0.8624 | 0.8235 | 2261447.7 | 2247822.6 | 1 |
| 398 | 2345 | 3,48 | Hexadecanoic acid methyl ester, n- | 949 | 949 | 112-39-0 | 1924 | 1923.1 | 0.9 | 0.6449 | 0.5999 | 1789190.4 | 1788151.8 | 0 |
| 409 | 2435 | 3,3 | 1,11-Undecanedioic acid, di(trimethylsilyl) ester | 825 | 836 | 106450-25-3 | 1996 | 1985 | 11 | 0.8886 | 0.7518 | 249803.5 | 252009.0 | -1 |
| 412 | 2450 | 2,12 | GLUCONIC ACID-6TMS | 908 | 908 | 34290-52-3 | 1990 | 1994.6 | 4.6 | 0.3146 | 0.2688 | 28635.4 | 27792.3 | 3 |
| 417 | 2470 | 4,36 | XANTHINE, N,O,O-TMS | 883 | 883 | 69-89-6 | 2011 | 2010.6 | 0.4 | 0.2953 | 0.2890 | 11591.6 | 12620.7 | -8 |
| 419 | 2485 | 3,31 | cis-9-Hexadecenoic acid, trimethylsilyl ester | 851 | 854 | 373-49-9 | 2017 | 2021 | 4 | 0.8334 | 0.8273 | 570138.1 | 561145.6 | 2 |
| 423 | 2490 | 2,16 | INOSITOL, scyllo-6TMS | 865 | 865 | 87-89-8 | 2021 | 2023.8 | 2.8 | 0.6503 | 0.6273 | 17637.9 | 16842.6 | 5 |
| 427 | 2490 | 3,5 | Heptadecanoic acid methyl ester, n- | 817 | 817 | 1731-92-6 | 2025 | 2024.8 | 0.2 | 0.2667 | 0.1594 | 17565.8 | 17114.8 | 3 |
| 430 | 2515 | 3,2 | HEXADECANOIC ACID-TMS | 910 | 924 | 55520-89-3 | 2043 | 2043.1 | 0.1 | 0.7556 | 0.7537 | 2265268.1 | 2224828.3 | 2 |
| 432 | 2545 | 4,63 | Salicyluric acid, bis(trimethylsilyl)- deriv. | 715 | 833 | 71428-95-0 | 2046 | 2066.4 | 20.4 | 0.3980 | 0.3214 | 22734.5 | 24218.3 | -6 |
| 436 | 2555 | 3,01 | N-ACETYL GLUCOSAMINE-MEOX O,O,O,O-TMS | 780 | 825 | 7512-17-6 | 2068 | 2072.6 | 4.6 | 0.1320 | 0.0849 | 10088.4 | 10487.2 | -4 |
| 438 | 2560 | 4,74 | 1H-Indole-3-propanoic acid, 1-(trimethylsilyl)-, trimethylsilyl ester | 747 | 785 | 55191-57-6 | 2089 | 2077.6 | 11.4 | 0.5293 | 0.5042 | 32595.5 | 30661.2 | 6 |
| 441 | 2575 | 2,46 | INOSITOL, myo-6TMS | 920 | 920 | 87-89-8 | 2087 | 2087 | 0 | 0.1583 | 0.1049 | 2544735.3 | 2440878.3 | 4 |
| 442 | 2580 | 0,3 | NAPROXEN-TMS, * | 959 | 959 | 22204-53-1 | 2085 | 2089.1 | 4.1 | 0.9174 | 0.9163 | 36948.8 | 13735.0 | **169** |
| 445 | 2590 | 3,43 | Uric acid (4TMS) | 887 | 887 | 69-93-2 | 2089 | 2098.8 | 9.8 | 0.6065 | 0.5434 | 1313910.5 | 1306437.3 | 1 |
| 446 | 2585 | 3,72 | Octadecadienoic acid methyl ester, 9,12-(Z,Z)-, n- | 941 | 941 | 112-63-0 | 2095 | 2095.3 | 0.3 | 0.5148 | 0.4426 | 443014.8 | 443216.5 | 0 |
| 447 | 2595 | 3,65 | Octadecenoic acid methyl ester, 9-(Z) | 953 | 953 | 1937-62-8 | 2106 | 2102.7 | 3.3 | 0.7517 | 0.7043 | 481716.8 | 486263.3 | -1 |
| 451 | 2615 | 2,78 | 1) HEPTADECANOIC ACID-TMS | 924 | 924 | 506-12-7 | 2142 | 2116.9 | 25.1 | 0.9734 | 0.9684 | 27281.2 | 27635.6 | -1 |
| 452 | 2620 | 3,1 | HEPTADECENOIC ACID, cis-10-TMS | 917 | 924 | 112-79-8 | 2120 | 2120.8 | 0.8 | 0.6273 | 0.5957 | 21187.5 | 21482.4 | -1 |
| 460 | 2640 | 2,27 | GLUCO-GULO-HEPTOSE-MEOX 6TMS_2 | 728 | 728 | 3146-50-7 | 2143 | 2135 | 8 | 0.5682 | 0.5252 | 55085.9 | 51443.9 | 7 |
| 463 | 2650 | 2,86 | 2) HEPTADECANOIC ACID-TMS | 945 | 945 | 506-12-7 | 2142 | 2142.9 | 0.9 | 0.6087 | 0.5853 | 66491.1 | 65409.5 | 2 |
| 468 | 2650 | 4,38 | 4-HYDROXY-HIPPURIC ACID-2TMS | 866 | 875 | 2482-25-9 | 2121 | 2144 | 23 | 0.4543 | 0.4637 | 5098.9 | 4749.6 | 7 |
| 474 | 2670 | 2,63 | Octadecane, 1-trimethylsilyloxy- | 817 | 826 | 18748-98-6 | 2152 | 2157.5 | 5.5 | 0.6249 | 0.2998 | 31183.6 | 32575.3 | -4 |
| 478 | 2700 | 4,08 | Gamma-Glutamyl-Leucine_2TMS | 614 | 772 | 2566-39-4 | 2178 | 2180.8 | 2.8 | 0.8988 | 0.8581 | 7374.2 | 7267.0 | 1 |
| 479 | 2725 | 0,2 | TRYPTOPHAN,N,O-TMS (2TMS) | 822 | 828 | 1033331-59-7 | 2202 | 2196.4 | 5.6 | 0.8711 | 0.7229 | 126364.2 | 125739.5 | 0 |
| 482 | 2720 | 3,44 | OCTADECATRIENOIC ACID, 6,9,12-(Z,Z,Z)-TMS | 836 | 836 | 506-26-3 | 2188 | 2195.1 | 7.1 | 0.7530 | 0.7354 | 6376.3 | 9828.2 | -35 |
| 484 | 2735 | 3,48 | OCTADECADIENOIC ACID, 9,12-(Z,Z)-TMS | 930 | 930 | 56259-07-5 | 2208 | 2206.8 | 1.2 | 0.3030 | 0.2732 | 2436954.3 | 2319504.1 | 5 |
| 485 | 2735 | 3,78 | TRYPTOPHAN,N,N,O-TMS (3TMS) | 928 | 928 | 55429-28-2 | 2208 | 2207 | 1 | 0.5859 | 0.2410 | 685005.5 | 649324.3 | 5 |
| 488 | 2745 | 3,34 | OCTADECENOIC ACID,-9-(Z)-TMS | 954 | 954 | 21556-26-3 | 2214 | 2214.7 | 0.7 | 0.5676 | 0.5574 | 2958168.3 | 2915312.1 | 1 |
| 495 | 2780 | 3,26 | OCTADECANOIC ACID-TMS | 924 | 924 | 18748-91-9 | 2244 | 2242.6 | 1.4 | 0.6282 | 0.6022 | 3460103.7 | 3435650.9 | 1 |
| 498 | 2800 | 4,3 | 5,8,11,14-Eicosatetraenoic acid, methyl ester, (all-Z)- | 887 | 889 | 2566-89-4 | 2231 | 2259.4 | 28.4 | 0.5202 | 0.4490 | 118993.9 | 114730.9 | 4 |
| 504 | 2840 | 3,11 | CYSTINE,N,N,O,O-TMS | 918 | 918 | 69688-44-4 | 2283 | 2290.5 | 7.5 | 0.3956 | 0.1528 | 75567.0 | 70294.3 | 8 |
| 517 | 2890 | 2,68 | Pseudo uridine (5TMS) | 812 | 817 | 53294-25-0 | NA | 2738.8 |  | 0.3357 | 0.1884 | 33180.4 | 32469.0 | 2 |
| 523 | 2930 | 2,77 | 1) EICOSATETRAENOIC ACID, 5,8,11,14-(Z,Z,Z,Z,)-TMS | 693 | 701 | 506-32-1 | 2364 | 2362.2 | 1.8 | 0.9472 | 0.9344 | 20713.3 | 20276.6 | 2 |
| 524 | 2930 | 3,29 | 2) EICOSATETRAENOIC ACID, 5,8,11,14-(Z,Z,Z,Z,)-TMS | 685 | 697 | 506-32-1 | 2364 | 2362.6 | 1.4 | 0.9645 | 0.9580 | 18140.5 | 17830.1 | 2 |
| 525 | 2930 | 3,56 | 3) EICOSATETRAENOIC ACID, 5,8,11,14-(Z,Z,Z,Z,)-(TMS) | 936 | 936 | 506-32-1 | 2364 | 2362.8 | 1.2 | 0.1078 | 0.0723 | 322095.0 | 304875.1 | 6 |
| 530 | 2940 | 3,62 | 4) EICOSATETRAENOIC ACID, 5,8,11,14-(Z,Z,Z,Z,)-TMS (-like) | 878 | 878 | 506-32-1 | 2364 | 2370.9 | 6.9 | 0.4002 | 0.3663 | 86898.3 | 77845.4 | 12 |
| 532 | 2965 | 2,82 | Myristic acid, 2,3-bis(trimethylsiloxy)propyl ester | 920 | 922 | 1188-73-4 | 2393 | 2390.3 | 2.7 | 0.4130 | 0.4141 | 26868.2 | 29607.0 | -9 |
| 534 | 2960 | 3,47 | 5) EICOSATETRAENOIC ACID, 5,8,11,14-(Z,Z,Z,Z,)-TMS (-like) | 834 | 839 | 506-32-1 | 2364 | 2386.8 | 22.8 | 0.1153 | 0.0963 | 82406.2 | 76058.6 | 8 |
| 536 | 2985 | 2,64 | myo-Inositol-2-phosphate (7TMS) | 859 | 886 | 33910-06-4 | 2429 | 2406.6 | 22.4 | 0.3297 | 0.2806 | 197316.4 | 191309.4 | 3 |
| 539 | 2990 | 3,43 | EIOCOSENOIC ACID, cis-11-TMS | 920 | 926 | 5561-99-9 | 2413 | 2411.7 | 1.3 | 0.4450 | 0.4252 | 66657.8 | 62470.7 | 7 |
| 543 | 3020 | 3,28 | EICOSANOIC ACID-TMS | 898 | 906 | 55530-70-6 | 2439 | 2437.6 | 1.4 | 0.7280 | 0.7256 | 36336.4 | 35061.8 | 4 |
| 544 | 3020 | 4,13 | URIDINE-4TMS | 718 | 720 | 58-96-8 | 2437 | 2438.4 | 1.4 | 0.2942 | 0.2538 | 39085.3 | 37434.3 | 4 |
| 547 | 3030 | 4,5 | 1) Docosahexaenoic acid methyl ester, 4,7,10,13,16,19-(Z,Z,Z,Z,Z,Z)-, n- | 932 | 934 | 2599-90-7 | 2456 | 2447.4 | 8.6 | 0.5988 | 0.5541 | 67899.0 | 66062.7 | 3 |
| 550 | 3050 | 4,1 | 2) Docosahexaenoic acid methyl ester, 4,7,10,13,16,19-(Z,Z,Z,Z,Z,Z)-, n- | 824 | 874 | 2599-90-7 | 2456 | 2464.4 | 8.4 | 0.3593 | 0.2848 | 9575.0 | 9360.5 | 2 |
| 562 | 3150 | 2,8 | 2-Monopalmitin trimethylsilyl ether | 829 | 831 | 53212-97-8 | 2576 | 2550.3 | 25.7 | 0.4788 | 0.4069 | 14826.3 | 15497.8 | -4 |
| 563 | 3150 | 4,21 | DOCOSAHEXAENOIC ACID-TMS | 939 | 939 | 6217-54-5 | 2552 | 2551.5 | 0.5 | 0.6047 | 0.5923 | 180846.7 | 173838.6 | 4 |
| 566 | 3165 | 3,52 | INOSINE-4TMS | 901 | 901 | 58-63-9 | 2562 | 2563.9 | 1.9 | 0.9451 | 0.9298 | 9239.2 | 7402.2 | 25 |
| 567 | 3170 | 4,09 | DOCOSAPENTAENOIC ACID-TMS | 945 | 945 | 24880-45-3 | 2571 | 2568.8 | 2.2 | 0.2695 | 0.2271 | 28882.5 | 27141.3 | 6 |
| 568 | 3190 | 2,97 | 1-MONOPALMITOYLGLYCEROL-2TMS | 880 | 882 | 1188-74-5 | 2587 | 2585.2 | 1.8 | 0.5873 | 0.5290 | 146069.0 | 152400.0 | -4 |
| 572 | 3220 | 3,52 | cis-13-Docosenoic acid, trimethylsilyl ester | 850 | 850 | 112-86-7 | 2608 | 2612.9 | 4.9 | 0.7016 | 0.7020 | 36418.2 | 39300.5 | -7 |
| 580 | 3245 | 3,31 | Docosanoic acid, trimethylsilyl ester | 817 | 821 | 74367-36-5 | 2638 | 2636.5 | 1.5 | 0.5845 | 0.5892 | 14825.0 | 13601.2 | 9 |
| 583 | 3295 | 2,4 | LACTOSE-MEOX 8TMS_1 | 893 | 893 | 42390-78-3 | 2675 | 2683.2 | 8.2 | 0.6864 | 0.6846 | 31237.1 | 30732.8 | 2 |
| 590 | 3345 | 1,99 | MALTOSE-MEOX 8TMS_1 | 893 | 893 | 19945-84-7 | 2722 | 2730.5 | 8.5 | 0.4737 | 0.3894 | 38921.2 | 37583.7 | 4 |
| 592 | 3370 | 3,35 | 9-Octadecenoic acid, 2-[(trimethylsilyl)oxy]-1-[[(trimethylsilyl)oxy]methyl]ethyl ester | 713 | 734 | 56554-42-8 | 2746 | 2755.6 | 9.6 | 0.4718 | 0.3639 | 164695.1 | 177174.8 | -7 |
| 594 | 3375 | 2,52 | LAMINARIBIOSE-MEOX 8TMS_2 | 907 | 907 | 34980-39-7 | 2752 | 2759.5 | 7.5 | 0.6709 | 0.5942 | 20646.4 | 20242.0 | 2 |
| 597 | 3395 | 3,02 | 1-MONOSTEAROYLGLYCEROL-2TMS | 919 | 919 | 123-94-4 | 2785 | 2779.1 | 5.9 | 0.3924 | 0.3318 | 82668.4 | 86730.6 | -5 |
| 603 | 3445 | 2,42 | 1) MALTITOL-9TMS | 862 | 878 | 585-88-6 | 2825 | 2827.4 | 2.4 | 0.2798 | 0.2386 | 634.1 | 621.0 | 2 |
| 607 | 3450 | 2,01 | 2) MALTITOL-9TMS | 812 | 863 | 585-88-6 | 2825 | 2832 | 7 | 0.2505 | 0.1852 | 2633.5 | 2472.6 | 7 |
| 620 | 3610 | 4,04 | GAMMA-TOCOPHEROL-(TMS) | 911 | 913 | 7616-22-0 | 2991 | 2994 | 3 | **0.0008** | **0.0009** | 9483.4 | 6495.3 | **46** |
| 626 | 3650 | 3,62 | 1-PALMITOYL-sn-GLYCERO-3-PHOSPHOCHOLINE-TMS derivative | 829 | 848 | 17364-16-8 | 3030 | 3035.4 | 5.4 | 0.1391 | 0.0916 | 879305.2 | 841576.0 | 4 |
| 632 | 3720 | 3,75 | 1) 7β-Hydroxycholesterol, bis(trimethylsilyl) ether | 813 | 820 | 33287-26-2 | 3124 | 3109.2 | 14.8 | 0.9804 | 0.9739 | 26093.6 | 26528.2 | -2 |
| 636 | 3755 | 4,41 | CHOLESTEROL-TMS | 938 | 938 | 1856-05-9 | 3150 | 3146.7 | 3.3 | 0.5503 | 0.2756 | 15586996.8 | 15401028.0 | 1 |
| 644 | 3845 | 4,27 | 2) 7β-Hydroxycholesterol, bis(trimethylsilyl) ether | 815 | 822 | 33287-26-2 | 3214 | 3234.1 | 20.1 | 0.8822 | 0.8332 | 40992.9 | 41878.7 | -2 |
| 657 | 3745 | 4,45 | ALPHA-TOCOPHEROL-(TMS) | 848 | 850 | 7695-91-2 | 3138 | 3136.2 | 1.8 | **0.0041** | **0.0018** | 28673.3 | 20639.9 | **39** |
|  | | | | | | | |  |  |  |  |  |  |  |
|  | | |  |  |  |  |  |  |  |  |  |  |  |  |
|  | |  |  |  |  |  |  |  |  |  |  |  |  |  |
|  | | | |  |  |  |  |  |  |  |  |  |  |  |
|  | | | |  |  |  |  |  |  |  |  |  |  |  |
|  | |  |  |  |  |  |  |  |  |  |  |  |  |  |

| RT - retention time, 1D/2D - 1^st^ or 2^nd^ GCxGC dimention, RI - retention index, DB - data base, NA - not available, GBM - glioblastoma, AU - arbitrary unit |
| --- |
| ^a^Match score value to NIST database |
| ^b^Obtained RI in this study |
| ^c^Deviation between measured RI in this study and RI in reference database |
| ^d^Percent of change in means relative to control, positive value indicate higher in case |
| * pharmaceutical drug |
